# Triplet therapy with venetoclax, FLT3 inhibitor and decitabine for *FLT3*-mutated acute myeloid leukemia

**DOI:** 10.1038/s41408-021-00410-w

**Published:** 2021-02-01

**Authors:** Abhishek Maiti, Courtney D. DiNardo, Naval G. Daver, Caitlin R. Rausch, Farhad Ravandi, Tapan M. Kadia, Naveen Pemmaraju, Gautam Borthakur, Prithviraj Bose, Ghayas C. Issa, Nicholas J. Short, Musa Yilmaz, Guillermo Montalban-Bravo, Alessandra Ferrajoli, Elias J. Jabbour, Nitin Jain, Maro Ohanian, Koichi Takahashi, Philip A. Thompson, Sanam Loghavi, Kathryn S. Montalbano, Sherry Pierce, William G. Wierda, Hagop M. Kantarjian, Marina Y. Konopleva

**Affiliations:** 1 Department of Leukemia, The University of Texas MD Anderson Cancer Center, Houston, TX USA; 2 Division of Pharmacy, The University of Texas MD Anderson Cancer Center, Houston, TX USA; 3 Department of Hematopathology, The University of Texas MD Anderson Cancer Center, Houston, TX USA

**Keywords:** Molecularly targeted therapy, Acute myeloid leukaemia

*FLT3* mutations occur in 20–35% patients with newly diagnosed (ND) acute myeloid leukemia (AML) and confer a higher risk of relapse and inferior overall survival (OS). Given modest benefit with first-generation multi-kinase inhibitors, second-generation FLT3 inhibitors (FLT3i) have been combined with low-intensity therapies (LIT) with encouraging results but are not curative^1–4^. Venetoclax with hypomethylating agent (HMA) has emerged as the new standard for older/unfit patients with AML^5^. Pre-clinical studies in *FLT3*^mut^ cell lines, primary samples, and xenografts have shown synergy between FLT3i’s and venetoclax through downregulation of Mcl-1 and Bcl-x_L_^6–9^. Clinical studies have demonstrated safety and activity of the combination of FLT3i and HMA with composite complete remission (CRc) rates of 65–80% and median OS 8.5–20 months^1,4,10^, as well as FLT3i and venetoclax which showed CRc rate of 85% in relapsed/refractory (R/R) *FLT3*^mut^ AML including in patients with prior FLT3i exposure^11^. We hypothesized that triplet therapy combining FLT3i, venetoclax, and HMA may further improve outcomes. Hence, we added FLT3i to our regimen of 10-day decitabine with venetoclax (DEC10-VEN) for *FLT3*^mut^ AML. We herein describe the first report of such a ‘triplet’ combination regimen for *FLT3*^mut^ AML.

This phase 2 trial (NCT03404193) enrolled ND patients with AML > 60 years and R/R patients >18 years. Patients needed to have ECOG performance status ≤3. Patients with favorable-risk cytogenetics and prior Bcl-2 inhibitor exposure were excluded. Patients received decitabine 20 mg/m^2^ IV for 10-days every 4–6 weeks for induction followed by decitabine for 5-days after CR/CRi, as described previously^12^. Venetoclax dose was 400 mg PO daily or equivalent (with azole co-administration). Reduction of venetoclax duration to <21 days per cycle was permitted in cases of persistent myelosuppression, after confirming ≤5% blasts or hypo/acellular marrow. Addition of FLT3i of clinician’s choice was allowed (Fig. S1). ND patients were admitted for the first cycle and R/R patients were admitted for the initial venetoclax ramp-up. Cytoreduction to WBC <10 × 10^9^/L was required prior to starting therapy and all patients received prophylaxis for tumor lysis syndrome, and antimicrobial prophylaxis.

Responses were graded per the IWG criteria for AML with adapted CRc criteria per the gilteritinib ADMIRAL and quizartinib QUANTUM-R studies^13,14^. The CRc included CR, CR with incomplete platelet recovery, and CR with incomplete hematologic recovery^13^. OS was measured from start of therapy until death or censored at last follow-up. Progression-free survival was defined from the time of response until relapse, death, or censored at last follow-up. Duration of response was determined from the time of response till relapse or censored at last follow-up or at the time of death without relapse. Measurable residual disease (MRD) was assessed on bone marrow (BM) specimens using 8-color multiparametric flow cytometry (FCM) validated to a sensitivity level of 0.01–0.1%. Negative results were considered valid if there had been acquisition of ≥200,000 events or ≥200 CD34+ myeloid precursors. A multiplex PCR-based test was used to detect *FLT3*-ITD or point mutations in codons 835/836 with an analytical sensitivity of 1% mutant reads in the background of wild-type reads. A targeted NGS panel was used to detect other mutations in *FLT3* and co-mutations in 80 other genes with an analytical sensitivity of 5% mutant reads in a background of wild-type reads.

Between April 30, 2018 and February 10, 2020, we treated 25 patients with *FLT3*^mut^ AML with this triplet combination. Twelve patients had ND AML and 13 patients had R/R AML (Table 1). The median age of the ND cohort was 70 years (IQR 69–78) and the R/R cohort was 52 years (interquartile range [IQR] 35–67). Median *FLT3* allelic ratio at enrollment in ND patients was 0.38 (IQR 0.17–0.45) and in R/R patients was 0.40 (IQR 0.32–0.52). The R/R cohort had received a median of 2 prior lines of therapies (IQR1–3) and 8 patients (57%) had received a prior FLT3i including sorafenib (*n* = 5), midostaurin (*n* = 2), gilteritinib (*n* = 1), and crenolanib (*n* = 1) with one patient having received two prior FLT3i. Four patients (29%) had received prior allogeneic hematopoietic stem-cell transplantation (HSCT).

**Table 1:** Baseline characteristics and outcomes of patients with *FLT3*^mut^ AML treated with FLT3 inhibitor, venetoclax, and 10-day decitabine.

	Newly diagnosed AML (*N* = 12)	Relapsed/refractory AML (*N* = 13)
*Baseline characteristics*		
Age, years	70 [69–78]	52 [35–67]
≥70 years	6 (50)	2 (15)
Male sex	4 (33)	10 (77)
ECOG performance status ≥2	4 (33)	3 (23)
Peripheral blood blasts, %	9 [3–51]	58 [35–70]
Bone marrow blasts, %	51 [46–75]	64 [54–68]
Diagnosis		
De novo	11 (92)	13 (100)
Secondary AML with AHD	1 (8)	0 (0)
ELN 2017 risk group		
Favorable	5 (42)	5 (38)
Intermediate	4 (33)	1 (7)
Adverse	3 (25)	7 (54)
ELN 2017 cytogenetic risk		
Favorable	0 (0)	0 (0)
Intermediate	12 (100)	9 (69)
Adverse	0 (0)	4 (31)
*FLT3*		
ITD high (≥0.5)	1 (8)	4 (31)
ITD low (<0.5)	7 (58)	6 (46)
TKD	3 (25)	1 (8)
ITD and TKD	1 (8)	1 (8)
Other	0 (0)	1 (8)^a^
Mutations		
* NPM1*	6 (50)	7 (54)
* IDH1/2*	4 (33)	1 (8)
* TP53*	0 (0)	2 (15)
* RUNX1*	2 (17)	2 (15)
* ASXL1*	3 (25)	1 (8)
* K/NRAS*	2 (17)	2 (15)
Prior therapies	0	1 [1–3]
FLT3 inhibitor		8 (62)
Hypomethylator (HMA)		2 (15)
Intensive chemotherapy (IC)		12 (92)
Stem-cell transplantation		4 (31)
*Outcomes*		
Composite complete remission rate (CRc)	11 (92)	8 (62)
CR	9 (75)	3 (23)
CRp	2 (17)	0 (0)
CRi	0 (0)	5 (38)
MRD negative		
by FCM	5/9 (56)	5/8 (63)
by PCR/NGS	10/11 (91)	7/7 (100)
No response	1 (8)	4 (31)
Aplasia	0 (0)	1 (8)
60-day mortality	0 (0)	1 (8)
Time to response, months	1.5 [1.3–2.7]	1.5 [1.0–2.4]
No. of cycles to response	1 [1–2]	2 [1–2]

All results expressed as no. (%) or median [interquartile range], unless specified. CR = complete remission with <5% blasts and absolute neutrophil count (ANC) ≥ 1 × 10^9^/L and platelet count ≥100 × 10^9^/L, CRp = achievement of all CR criteria except for platelet recovery (platelet count <100 × 10^9^/L), CRi = CR with incomplete hematologic recovery = achievement of all CR criteria except for hematologic recovery with residual neutropenia (ANC < 1 × 10^9^/L) with or without RBC/platelet transfusion independence; aplasia was defined as inevaluable bone marrow sample due to cellularity <10%.

*ECOG* Eastern Cooperative Oncology Group, *AHD* antecedent hematological disorder, *ELN* European LeukemiaNet, *MRD* minimal residual disease, *FCM* flow cytometry, *PCR* polymerase chain reaction, *NGS* next-generation sequencing.

^a^One patient had FLT3 S749L variant.

FLT3i used along with DEC10-VEN in the ND cohort included gilteritinib (*n* = 5), sorafenib (*n* = 5), and midostaurin (*n* = 2), and in the R/R cohort included sorafenib (*n* = 5), gilteritinib (*n* = 5), and midostaurin (*n* = 3). Median dose and duration of FLT3i during cycle 1 for sorafenib was 400 mg twice daily (BID; IQR 400–400) for 15 days (IQR 14–28), for midostaurin was 50 mg BID (IQR 50–50) for 15 days (IQR 14–21) and for gilteritinib was 120 mg daily (IQR 120–120) for 14 days (IQR 14-continuous). For subsequent cycles, the median dose and duration of sorafenib was 400 mg BID (IQR 400–400) for 14 days (IQR 14-continous), for midostaurin was 50 mg BID (IQR 50–50) daily continuously (IQR 28-continuous) and for gilteritinib was 120 mg (IQR 80–120) daily continuously (IQR 24-continuous). Details of reductions in FLT3 inhibitor dose and venetoclax duration are mentioned in the supplement and Fig. S2. In ND patients, delay in starting subsequent cycle beyond 42 days occurred in 18 (43%) out of 42 evaluable cycles. Among R/R patients, such delay occurred in 7 (37%) out of 19 evaluable cycles. The last ongoing cycle at the time of data cut-off was not included in this analysis.

In ND patients, the CRc rate was 92% with MRD negativity by FCM in 56% and by PCR/NGS in 91% of responders (Table 1). In R/R AML the CRc rate was 62% with MRD negativity rate by FCM in 63% and by PCR/NGS in 100% of responders. Among 8 patients with R/R AML and prior exposure to a FLT3i the CRc rate was 63%, with *FLT3* PCR negativity in 4 out of 4 responding patients tested.

The 60-day mortality was 0% in ND patients and 7% (*n* = 1) in R/R patients. There were 50 non-hematologic adverse events (AE) in 25 patients, at least possibly related to study regimen, with most frequent grade 3/4 AEs being febrile neutropenia in 40% patients (*n* = 10), infections with grade 3/4 neutropenia in 36% patients (*n* = 9), infection with absolute neutrophil count (ANC) ≥ 1.0 × 10^9^/L in 32% patients (*n* = 8), and tumor lysis syndrome in 16% patients (*n* = 4, Table S1). In responding patients with ND AML, the median time to ANC recovery to ≥0.5 × 10^9^/L after cycle 1 was 44 days and after subsequent cycles was 38 days (Fig. 1a); and median time to platelet recovery to ≥50 × 10^9^/L after cycle 1 was 34 days and <50% patients had platelet count drop below 50 × 10^9^/L during subsequent cycles (Fig. 1b). In responding patients with R/R AML, median time to ANC recovery after cycle 1 was 38 days and after subsequent cycles was 47 days. The median cycle durations in ND AML for cycle 1 and cycle 2 were 46 days (IQR 43–52) and 42 days (IQR 42–69), respectively, and in R/R AML were 32 days (IQR 30–44) and 47 days (IQR 38–61), respectively.

**Fig. 1:**
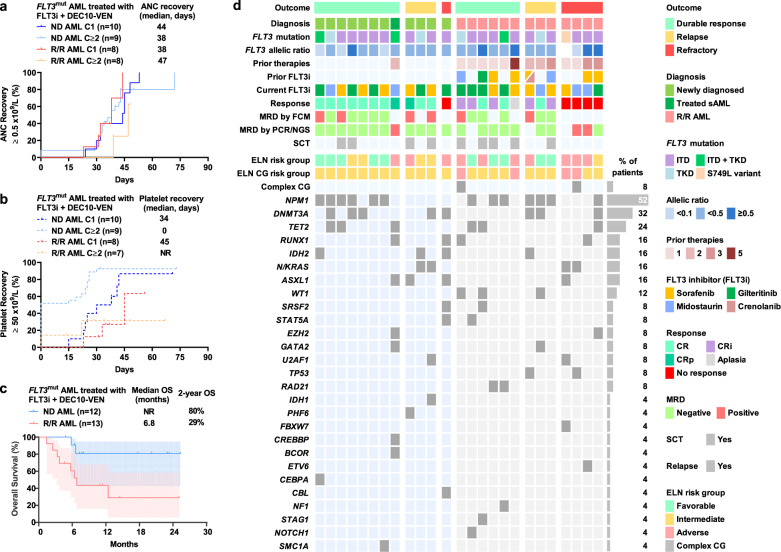
Outcomes of patients with newly diagnosed (ND) and relapsed/refractory (R/R) acute myeloid leukemia with *FLT3*^mut^ treated with FLT3 inhibitor (FLT3i), venetoclax, and 10-day decitabine (DEC10-VEN). **a** Absolute neutrophil count (ANC) recovery to ≥0.5 × 10^9^/L, **b** platelet count recovery to ≥50 × 10^9^/L, and **c** overall survival (OS), and **d** mutational landscape of all patients. NR not reached, MRD measurable residual disease, FCM flow cytometry, multiplex PCR polymerase chain reaction, PCR-based NGS next-generation sequencing.

After a median follow-up 14.5 months (95% CI 7.7–23.0) the median OS in ND patients was not reached with 2-year OS of 80%, and in R/R patients was 6.8 months (Fig. 1c). The 18-month progression-free survival in ND and R/R AML was 59% and 58%, respectively (Fig. S3). The median duration of response was not reached in either ND or R/R patients (range 0.8–24.3 months; Fig. S4). Four ND patients underwent HSCT after response and two patients received maintenance with sorafenib (*n* = 1) and crenolanib (*n* = 1). Five R/R patients underwent HSCT and one patient received maintenance with decitabine and sorafenib afterward. The 2-year OS in patients undergoing HSCT for ND and R/R patients was 100% and 53%, respectively (Fig. S5). A detailed mutational landscape is shown in Fig. 1d.

Two ND patients and eight R/R patients have died. In the ND cohort, two patients died in CR/CRp due to pneumonia with grade 1 neutropenia (*n* = 1, after elective discontinuation after cycle 5) and unknown reason (*n* = 1). Among R/R patients, four patients who were refractory to this regimen died from infectious complications (*n* = 3) and of unknown reason (*n* = 1); three responding patients died of unknown reasons after relapse and one patient with aplasia died from infectious complications after HSCT. Infections contributing to death, regardless of attribution, in one ND patient and three R/R patients included pneumonia due to *Stenotrophomonas maltophila* (*n* = 1) and unknown pathogen (*n* = 3). Among five responding patients who died, there were no deaths known to be possibly, probably, or definitely related to the study regimen. The causes of death in these five patients included pneumonia due to unknown pathogen (*n* = 1) and unknown reason (*n* = 4). Among five patients without a response who died, one death was possibly related to study regimen due to pneumonia in the setting of persistent pancytopenia and aplastic bone marrow with <5% cellularity 16 days prior to death. Twenty patients discontinued treatment for reasons including HSCT (*n* = 9, 36%), resistant disease (*n* = 4, 16%), relapse (*n* = 2, 8%), withdrawal of consent (*n* = 2, 8%), completion of treatment (*n* = 1, 4%), toxicity (glomerulonephritis, *n* = 1, 4%), and death in CRp (*n* = 1, 4%).

The outcomes in ND patients with 2-year OS of 80% compare favorably with prior reports of sorafenib, quizartinib, or gilteritinib with LITs which have yielded ORR of 67–92% and median OS of 8.3–18.6 month^2–4^. The outcomes in salvage setting with CRc rate of 62% and median OS of 6.8 months are comparable to non-venetoclax based doublet regimens of sorafenib, midostaurin, or quizartinib with LITs which have yielded overall response rates (ORR) of 26–83% and median OS of 5.1–11.3 months^1,3^. In comparison, venetoclax with gilteritinib has shown a CRc of 85% in R/R *FLT3*^mut^ AML^11^.

The rationale for selecting the 10-day regimen over 5-days of decitabine as the backbone include previous pharmacodynamic data suggesting better efficacy of the 10-day regimen and high response rates of 40–64% in AML with unfavorable risk cytogenetics^12,15^. Although there were delays in blood count recovery, the rates of neutropenic fever in 40% patients and infections with grade 3/4 neutropenia in 36% patients were comparable to the 30% rate of neutropenic fever and 64% rate of grade 3/4 infections with azacitidine and venetoclax^5^. Deaths due to infectious etiology occurred in one ND patient (8%) which was comparable to 7% infection-related deaths noted with frontline HMA and venetoclax^16^. Hence, without prospective studies, it is difficult to speculate about the risk-benefit ratio of using a 5-day decitabine or 7-day azacitidine regimen as the backbone for such triplet combinations. Future trials need to establish the optimal schedule of venetoclax and FLT3i doublets and triplets to minimize toxicity and maximize efficacy.

For *FLT3*^mut^ patients who are candidate for LITs, we are currently using second-generation FLT3i gilteritinib 80 mg daily, based on similar efficacy to 120 mg dose^17^, and using a cycle 1 day-14 bone marrow to evaluate for response or marrow ablation to determine withholding of venetoclax to promote for earlier ANC recovery in first cycle. This may allow for a longer ‘venetoclax holiday’ from day-14 onward and potentially improve myelosuppression with such triplet therapy. After achievement of response, we recommend continuous daily dosing of FLT3i and decreasing duration of venetoclax to 14–21 days based on count recovery period in cycle 1 and adding myeloid growth factors as needed to minimize duration of neutropenia. Other trials testing similar triplet combinations of HMA, venetoclax with quizartinib (NCT03661307) and gilteritinib (NCT04140487) in ND and R/R AML are currently ongoing and will determine the optimal combinatorial approach for these agents. While the doublet combination of FLT3i with venetoclax has shown high CRc rate of 85% in R/R *FLT3*^mut^ AML, we believe that triplet therapy with the addition of an HMA may offer broader activity and prolong responses and survival by eliminating other subclones and preventing secondary resistance^11,18^. This will need to be balanced against the potential increased myelosuppression with such triplet regimens. Continued accrual and longer follow-up of these trials will hopefully provide more answers and help optimize the selection of the doublets or triplets in specific patient populations.

Some limitations of our study include the use of different FLT3i across different generations which may limit extrapolation of our results. Many patients at our center travel from far locations and choose to receive portion of their care closer to home. Consequently, we could not ascertain causes of deaths in some patients.

In conclusion, triplet therapy with FLT3i, venetoclax, and decitabine is safe and an excellent frontline option for older patients with ND *FLT3*^mut^ AML, and effective for R/R AML. Transition to HSCT and post-transplant maintenance with FLT3i may offer further improvement in long-term outcomes.

## Supplementary information

Supplemental appendix

Figure with individual panels as requested
