# Supplementary material for: Triplet therapy with venetoclax, FLT3 inhibitor and decitabine for FLT3-mutated acute myeloid leukemia
Source: Blood Cancer J. 2021 Feb 1;11(2):25. doi: 10.1038/s41408-021-00410-w (PMC7873265; doi:10.1038/s41408-021-00410-w)
Supplement: Supplementary file 1 — Supplemental appendix [file 41408_2021_410_MOESM1_ESM.pdf]

SUPPLEMENTAL APPENDIX

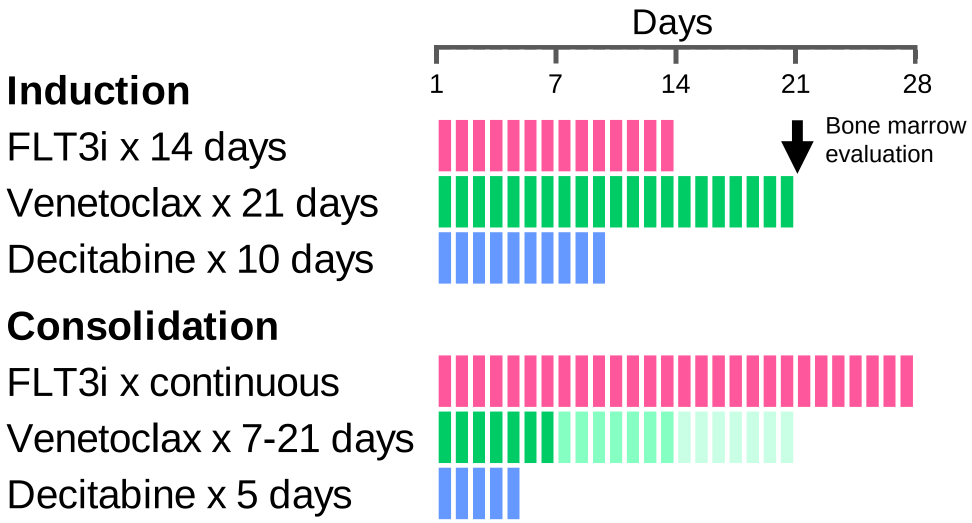

Fig. S1. Dosing schema – During induction, patients received decitabine 20 mg/m^2 for 10-days, FLT3 inhibitor daily for 14 days, and venetoclax 400 mg (or equivalent with azoles) daily. Bone marrow evaluation was done on cycle 1 day 21. If bone marrow evaluation showed blast clearance (<5%) then venetoclax was held to enable peripheral blood count recovery. If bone marrow evaluation revealed hypocellularity or aplasia, bone marrow aspiration was repeated after blood count recovery, or between day 28 to 42. During consolidation, patients received decitabine for 5 days, FLT3 inhibitor daily continuously, and venetoclax daily. Venetoclax duration could be reduced to <21 days to up to 7 days in cases of myelosuppression. Cycle length was 4 to 6 weeks.

FLT3 inhibitor dose reductions

In the newly diagnosed AML, 1 patient receiving gilteritinib and one receiving sorafenib were reduced to 80 mg and 400mg daily, respectively during induction. For cycles 2 and beyond, all patients treated with gilteritinib received a reduced dose of 80 mg daily. Among the 15 courses including sorafenib, 4 had doses reduced to 200-400mg daily. Four courses included midostaurin, of which 1 was dose reduced to 50 mg daily.

In patients with relapsed/refractory AML, 1 patient receiving gilteritinib and 1 receiving sorafenib had a dose reduction of 80 mg daily and 400 mg daily, respectively. One patient receiving sorafenib had a dose reduction to 400 mg per day after 42 days of continuous dosing of 800 mg per day while awaiting stem-cell transplantation. During cycles 2 and beyond, the 3 courses with sorafenib and 1 course with midostaurin were at full dose of 800 mg and 100 mg daily, respectively. Two of the 12 courses with gilteritinib were reduced to 80 mg daily.

Reduction in venetoclax duration

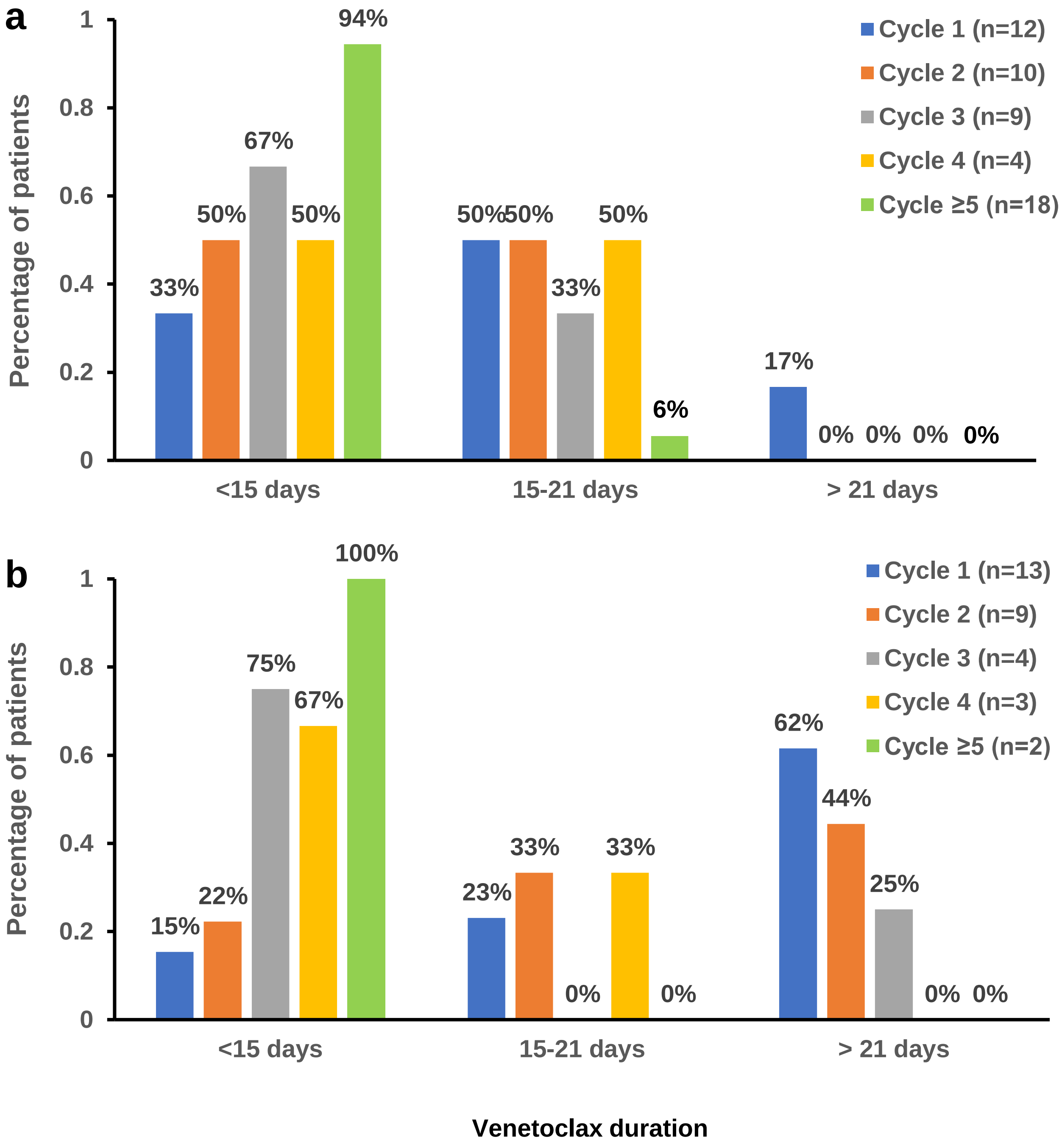

Fig. S2. Duration of venetoclax in combination with FLT3 inhibitor and 10-day decitabine in patients with **a.** newly diagnosed, and **b.** relapsed or refractory *FLT3*^{mut} acute myeloid leukemia.

Table S1. Treatment-emergent adverse events at least possibly related to study regimen of FLT3 inhibitor, venetoclax, and decitabine in *FLT3*^{mut} acute myeloid leukemia

**Treatment-emergent adverse events**	**Grade 1/2**	**Grade 3**	**Grade 4**
		**n (%)**	
Renal failure	..	..	1 (4)
Febrile neutropenia (ANC <1.0 x 10^9/L)	..	10 (40)	..
Infection with ANC >1.0 x 10^9/L	..	9 (36)	..
Infection with ANC <1.0 x 10^9/L[1]	1 (4)	8 (32)	..
Tumor lysis syndrome	2 (8)	2 (8)	..
Mucositis	3 (12)	1 (4)	..
Hyperbilirubinemia	..	1 (4)	..
Constipation	2 (8)	..	..
Diarrhea	2 (8)	..	..
Nausea	2 (8)	..	..

ANC = absolute neutrophil count. 1. These infections with ANC <1.0 x 10^9/L included pneumonia (n=6), cellulitis (n=2) and upper respiratory tract infection (n=1)

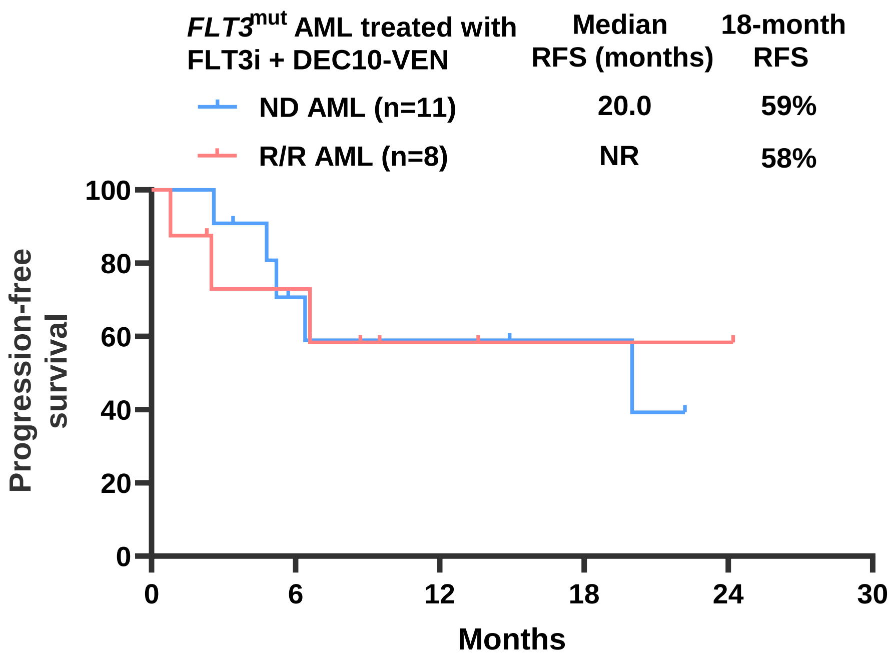

Fig. S3. Progression-free survival of patients with newly diagnosed (ND) and relapsed/refractory (R/R) acute myeloid leukemia (AML) with *FLT3*^{mut} treated with FLT3 inhibitor (FLT3i), venetoclax, and 10-day decitabine (DEC10-VEN).

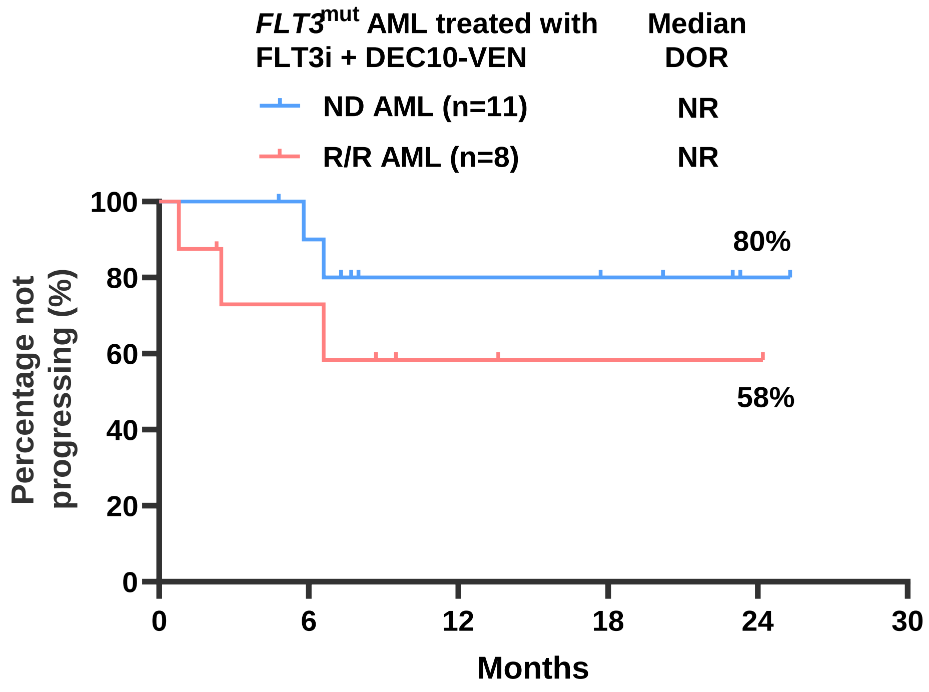

Fig. S4. Duration of response in patients with newly diagnosed (ND) and relapsed/refractory (R/R) acute myeloid leukemia (AML) with *FLT3*^{mut} treated with FLT3 inhibitor (FLT3i), venetoclax, and 10-day decitabine (DEC10-VEN). NR = not reached.

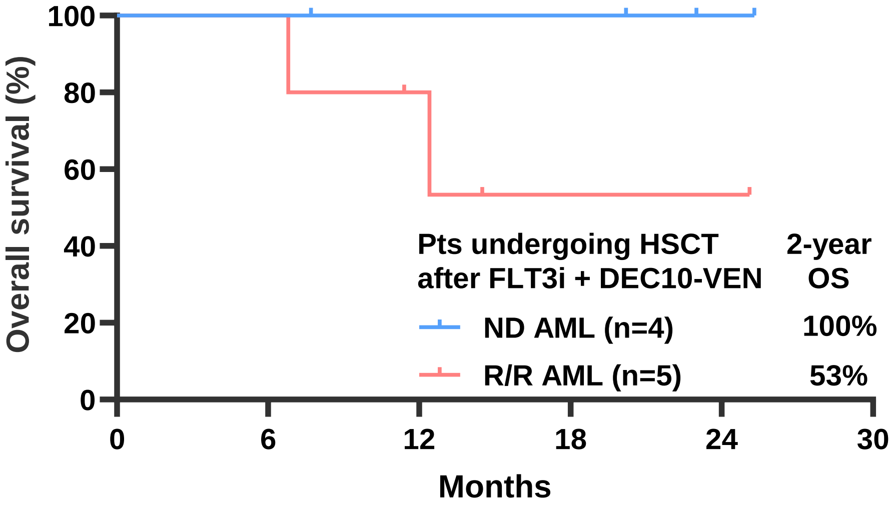

Fig. S5. Overall survival (OS) of patients (pts) with *FLT3*-mutant acute myeloid leukemia undergoing hematopoietic stem-cell transplantation (HSCT) after achieving response with FLT3 inhibitor (FLT3i), venetoclax and 10-day decitabine (DEC10-VEN).
