# Supplementary figures and images for: Triplet therapy with venetoclax, FLT3 inhibitor and decitabine for FLT3-mutated acute myeloid leukemia
Source: Blood Cancer J. 2021 Feb 1;11(2):25. doi: 10.1038/s41408-021-00410-w (PMC7873265; doi:10.1038/s41408-021-00410-w)

## Slide 1
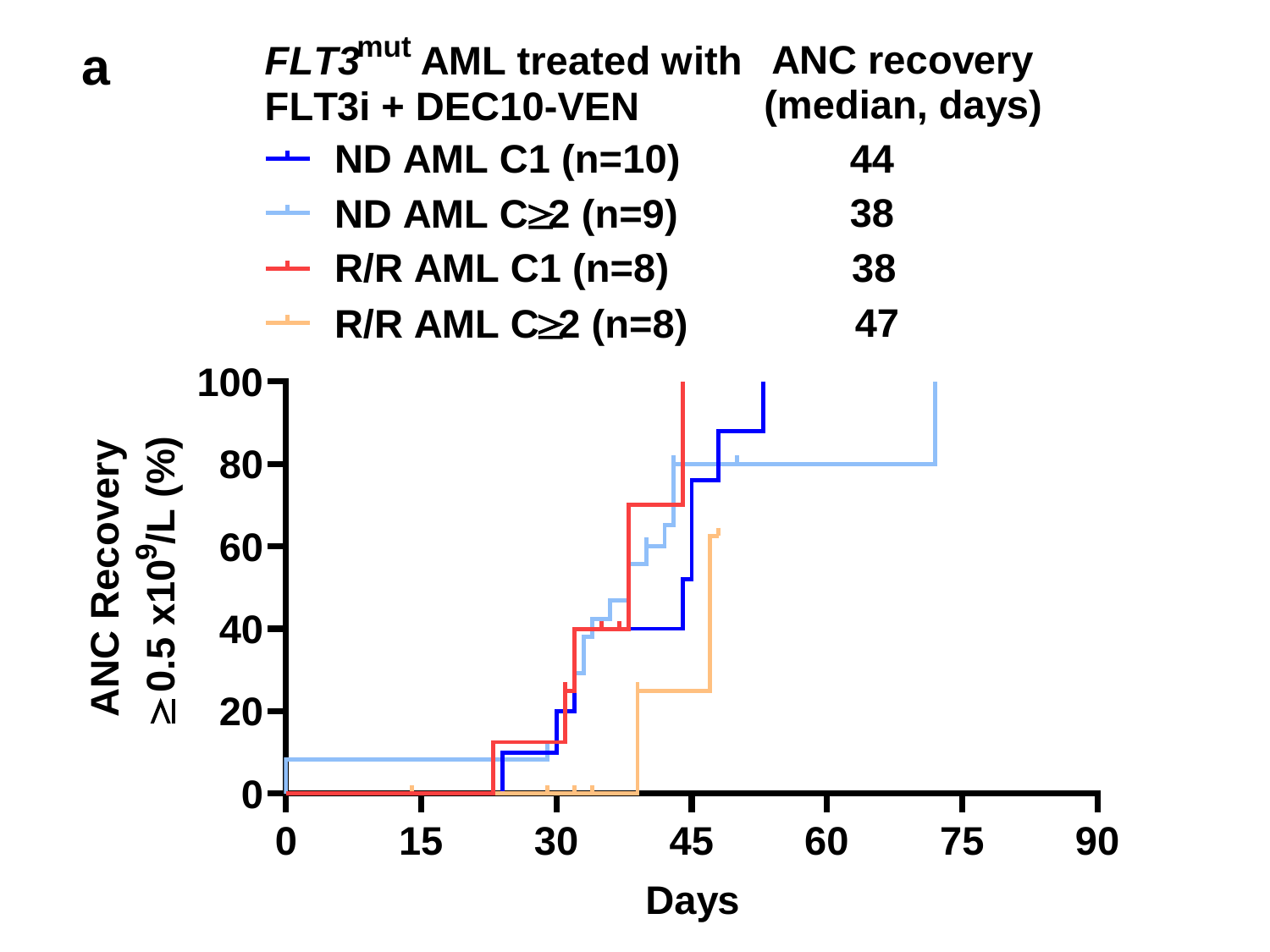

## Slide 2
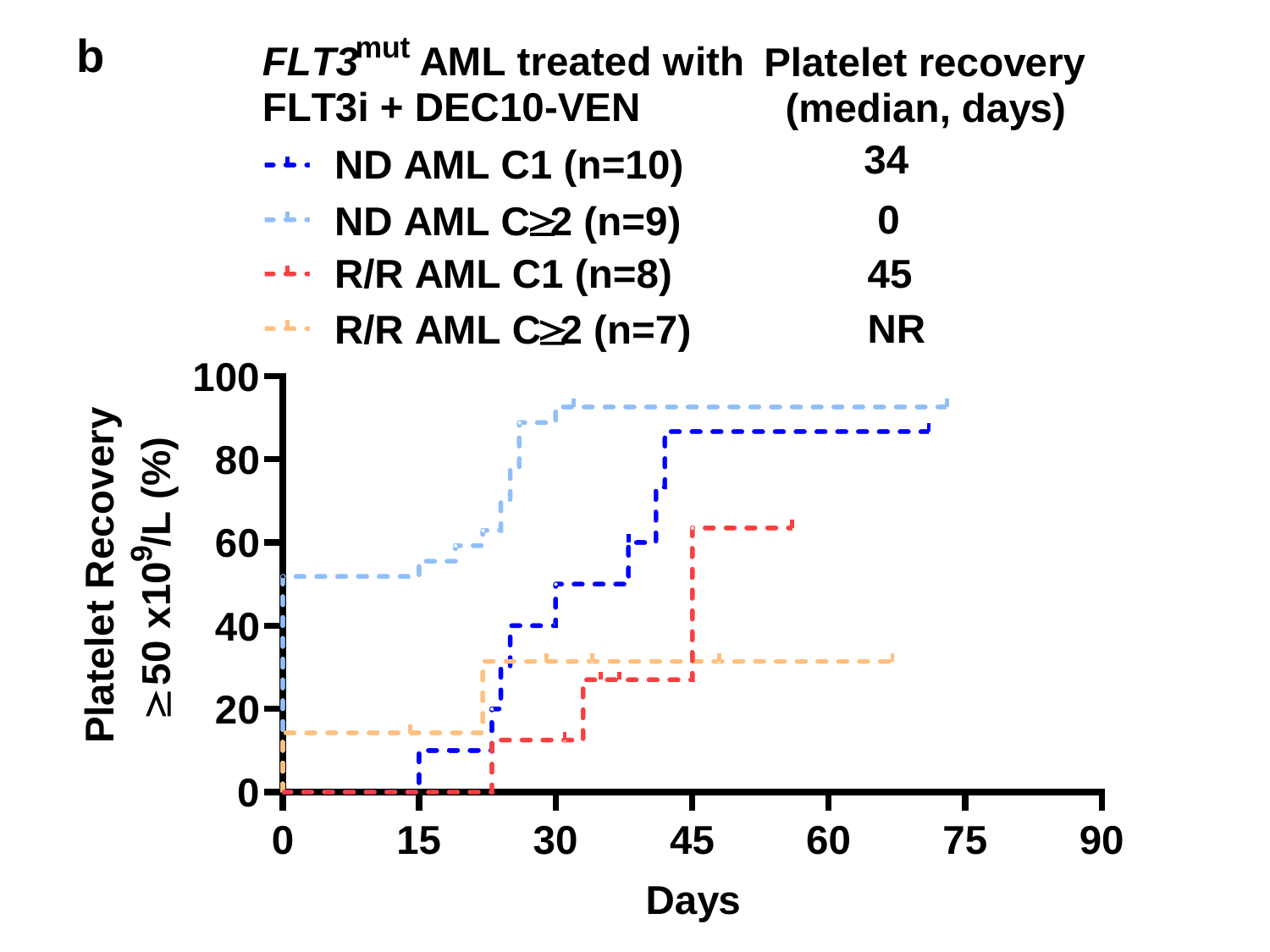

## Slide 3
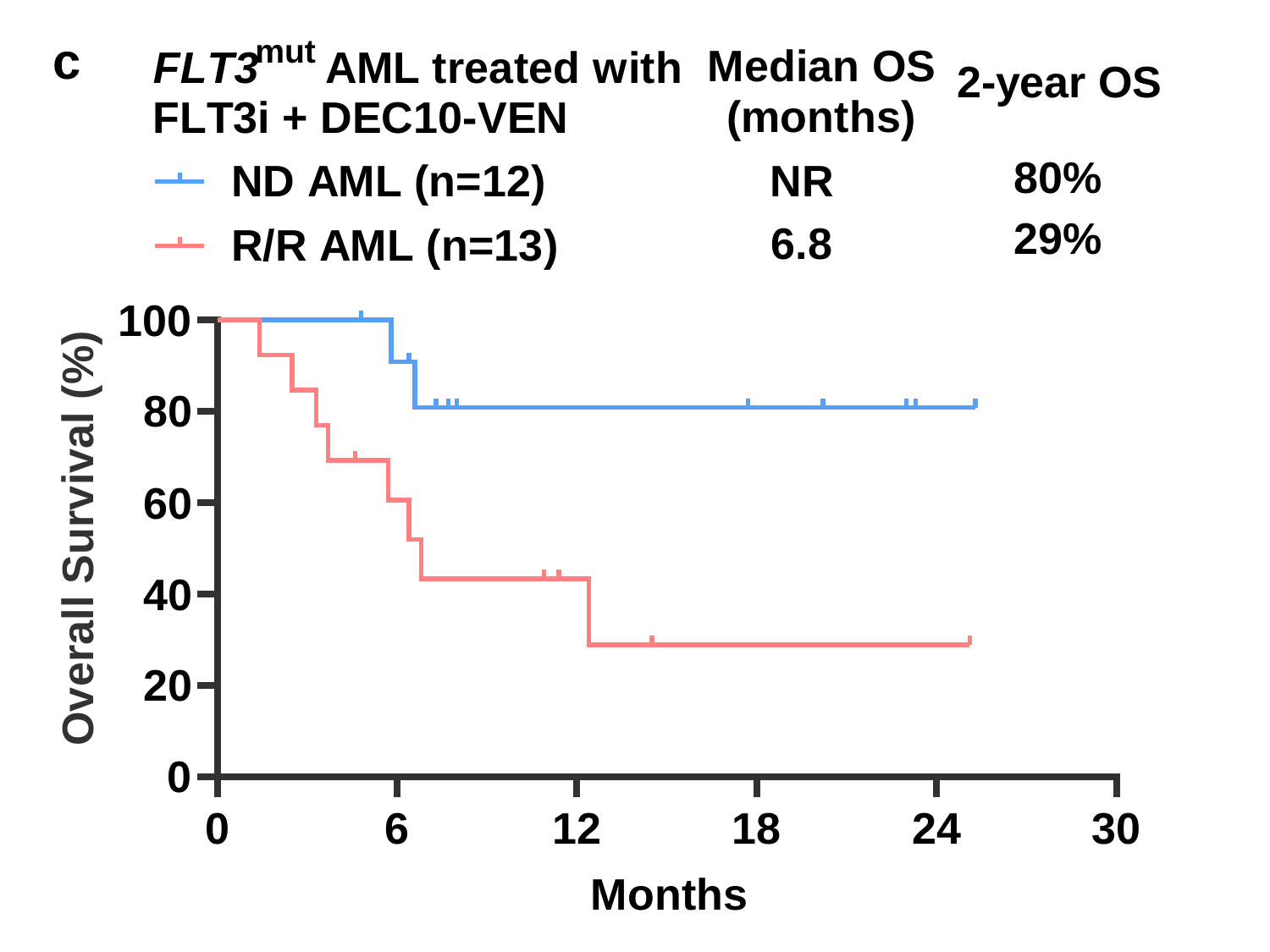

## Slide 4
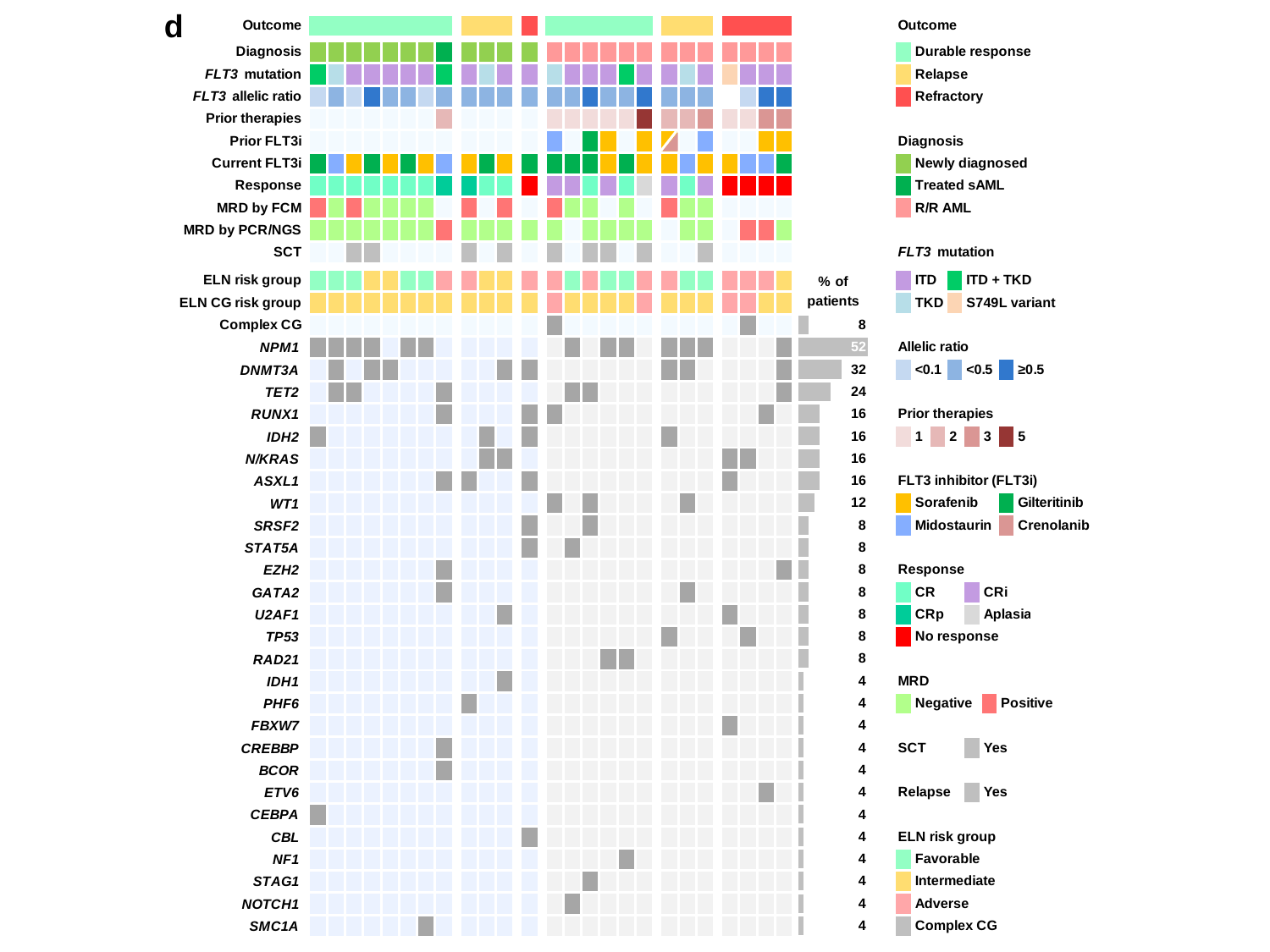

d

Supplement: Supplementary file 2 — Figure with individual panels as requested [file 41408_2021_410_MOESM2_ESM.pptx]
